# Measuring maternal improvement through multi-stakeholder engagement: Documenting developments in the dynamic programmatic context of the global COVID-19 pandemic through outcome harvesting

**DOI:** 10.7189/jogh.13.06016

**Published:** 2023-06-09

**Authors:** Jewel Gausman, Ana Langer, R Rima Jolivet

**Affiliations:** Department of Global Health & Population, Harvard T.H. Chan School of Public Health, Boston, Massachusetts, USA

## Abstract

**Background:**

To bolster country efforts towards meeting the targets and strategies laid out in WHO’s report “Strategies toward ending preventable maternal mortality” (EPMM), a series of seven consultations, known as National Dialogues, were conducted to better understand national priority areas for the improvement of maternal health and to support the adoption and use of EPMM indicators at the national level. The last Dialogue was conducted in March 2020, as the COVID-19 pandemic was beginning to have global impacts. We aimed to explore the circumstantial challenges and opportunities that countries have encountered in meeting the specific stakeholder commitments made in each country by National Dialogue participants during the COVID-19 pandemic.

**Methods:**

We based our study methodology on outcome harvesting, a qualitative approach that examines how incremental change contributes towards achieving a specified outcome. It collects evidence on what has changed and then works backwards to determine whether and how a programme or intervention led to the observed changes. We collected data from 20 participants in five countries (Bangladesh, India, Mexico, Nigeria, and Pakistan) through key informant interviews and focus group discussions. We analysed the data through inductive coding focused on emergent themes.

**Results:**

The onset of the global COVID pandemic overturned plans and upended health systems, bringing new opportunities in some countries and halting progress towards the agenda outlined in the National Dialogue elsewhere. Participants identified adaptations that facilitated continued progress, such as shifting the locus of advocacy and activity from national to sub-national focal areas, catalytic changes in response to the crisis (including the development and improvement of digital communication and data technology), and increased awareness of the importance of identified priorities (including a human rights approach to maternal health).

**Conclusions:**

Our data suggest that the priorities for maternal health system performance to drive improvement toward ending preventable maternal deaths and the advocacy commitments designed to increase the relevance of upstream policy and health system-level determinants of maternal health and survival have retained their urgency during the COVID-19 pandemic.

Maternal mortality has declined globally over the last several decades, however, progress has fallen short of expectations and significant disparities persist. One target of the Sustainable Development Goal (SDG) 3 is to reduce the global maternal mortality ratio to less than 70 per 100 000 live births by 2030. As of 2015, 25 countries had a maternal mortality ratio greater than 420 per 100 000 live births [1]. Among the ten countries with the highest MMRs in 2017, all but Afghanistan are located in sub-Saharan Africa, and all experienced average annual rates of reduction of less than five per cent between 2000 and 2017, compared to 38% across countries in sub-Saharan Africa and 52% in Central Asia [2]. Achieving SDG 3 will require continued global investment and prioritisation of maternal health research, programmes, and policy. A global systematic analysis found that, while haemorrhage was the leading cause of maternal death globally between 2003 and 2000, indirect causes accounted for more than a quarter of maternal deaths [3]. To accelerate progress in reducing preventable maternal deaths, countries must push past strategies that primarily focus on the major direct causes of maternal mortality and target the upstream determinants that may be contributing to plateaus in progress.

In 2015, the WHO released the “Strategies toward ending preventable maternal mortality” (EPMM Strategies) report, outlining global targets and strategies for reducing maternal mortality in the 2015-2030 SDGs era [4], focused on human rights and system performance to eliminate disparities in access, quality, and outcomes of maternal care both within and between countries. To reach these ambitious targets, the strategies highlight 11 EPMM key themes grounded in fundamental human rights principles of equity, non-discrimination, transparency, participation, and accountability. They represent the full, broad spectrum of determinants of maternal health and survival, including social/structural, political, economic, and health system-level determinants. A comprehensive monitoring framework for each theme was developed to track national and global progress in improving maternal health [5,6].

The Women & Health Initiative of the Harvard T. H. Chan School of Public Health launched the “Improving Maternal Health Measurement Capacity and Use” (IMHM) project to strengthen the EPMM framework’s indicators and use. During the project, seven consultations (known as National Dialogues) were conducted to better understand national priority areas for improvement related to maternal health and to support the adoption and use of EPMM indicators in national-level monitoring frameworks to drive improvement in those self-identified priority areas. Each National Dialogue included approximately 40-50 stakeholders representing expertise and commitment in each of the 11 EPMM key themes, including representatives from the Ministry of Health, UN and donor agencies, development partners, civil society advocates, and others from outside the health sector. The National Dialogues took place in seven countries: Bangladesh (February 2019), Cote d’Ivoire (November 2018), India (April 2019), Kenya (July 2018), Mexico (July 2019), Nigeria (March 2020), and Pakistan (October 2019), with each country facing a different array of challenges in relation to addressing the proximate and distal drivers of maternal mortality.

The National Dialogues aimed to identify local priorities and support efforts to improve the availability and use of robust monitoring data for advocacy so as to advance maternal health and enhance policy, programmatic and resource-related decision-making. As the National Dialogues were being completed, country participants agreed on specific actions and made commitments based on the identified priority issues.

During the last National Dialogue in Nigeria in March 2020, COVID-19 began impacting countries worldwide. Given the complexity of advancing the maternal health measurement agenda during a global pandemic, we believe that there may have been important similarities in both the challenges faced and the lessons learned across countries. While we did not expect any country to have fully achieved all priorities and commitments set during the National Dialogues, as progress towards them was greatly impacted by the global pandemic, we believe that a cross-country synthesis of how the COVID-19 pandemic either hampered or accelerated achievements may help with identifying lessons learned regarding the impact of global and national crises that compete for attention and resources on the progress of previously adopted agendas

With this study, we aimed to examine country-level developments, describe country experiences, and identify the unique and common challenges countries have faced in meeting the National Dialogues’ objectives and the specific stakeholder commitments made and advocacy targets identified in each country by National Dialogue participants during the COVID-19 pandemic. As the pandemic must have affected the visibility or focus on maternal health and/or measurement plans, we sought to explore the ways and directionality in which it had done so.

## METHODS

We based our study methodology on outcome harvesting, a qualitative approach that examines how incremental change contributes towards achieving a specified outcome [7]. At a basic level, it collects evidence on what has changed and then works backward to determine whether and how a programme or intervention led to the observed changes. While traditional monitoring and evaluation approaches compare expected vs achieved outcomes, outcome harvesting is better suited for dynamic programmatic contexts (where objectives and pathways to progress are unpredictable and not clearly or easily defined) and for complex programmes (where incremental change is important to capture). For example, a traditional indicator may measure the number of policies changed in a country; however, tracking this indicator quantitatively does not say anything about the multiple successes that had to be achieved along the way, which may be key in cases where substantial movement was made towards changing a policy, but the change had not yet occurred [8]. The overarching goal of outcome harvesting is not to seek sole attribution for the outcome, but to understand how an actor contributed it. Outcome harvesting seeks to understand how actors realise these changes through inspiration, support, facilitation, persuasion, or pressure [9]. Outcomes are broadly defined as any relevant change in behaviour, relationships, actions, activities, policies, or practices.

### Study participants

Data sources related to the developments identified in Step 1 were key informant interviews (KIIs) and focus group discussions (FGDs). A total of 16 key KIIs and two FGDs were conducted including four participants each (**Table 1**).

**Table 1:** Number of study participants from each country and data collection modality

Country	Participants	Modality
Bangladesh	3	3 KIIs
India	6	2 KIIs,1 FGD
Mexico	7	3 KIIs, 1 FGD
Nigeria	4	4 KIIs
Pakistan	3	3 KIIs

KII – key informant interview, FGD – focus group discussion

### Data collection

Our methodology reflected the sequence and substance of the steps of outcome harvesting, condensed to six steps for feasibility reasons. We prepared a semi-structured interview guide that outlined each step. Interviews were conducted by a trained facilitator who had previously been involved at the international level in planning the National Dialogues and was thus known by most of the participants.

#### Step 1: Designed the outcomes for harvest

The outcome harvest focused on understanding how the National Dialogues contributed to country-level developments toward meeting the specific objectives set by participating stakeholders and how the COVID-19 pandemic affected the priorities identified in each country or influenced the developments that took place toward addressing them. Developments were defined as any positive change in behaviour, relationships, actions, activities, policies, or practices relevant to the National Dialogue objectives or ensuing commitments in each country. We further intended to understand the development’s overall significance while uncovering other dimensions that led to change in the priority area, including the policy context and contributions of other actors or events and their intersection with the COVID-19 context.

#### Step 2: Gather data and draft outcome descriptions

The research team collected information about developments and how change agents (the actors/organisations that influence a development) contributed to achieving it.

Each FGD followed the process specified below:

− First, the facilitator reviewed the dialogue objectives, priorities, action items, and commitments for each country that were agreed upon in the National Dialogue.
− The facilitator discussed developments already identified in relation to those items and asked the participants to provide additional detail including the development’s significance and how specific actors contributed to the change (such as through facilitating, supporting, advocacy, etc.). Contributions could be direct or indirect, partial, or whole, intended or not.
− The facilitator asked for additional details related to any contributions, such as when they occurred and whether any other actors were involved.
− The facilitator asked participants whether any other outside actors contributed and how, whether COVID-19 or any other external events may have facilitated the change.

#### Step 3: Formulate outcome descriptions

The research team synthesised information from the KIIs and FGDs and developed draft outcome descriptions, which focused on who changed what, when, where, and how.

#### Step 4: Substantiate

The study team looked for convergence between one or more independent responses, corroborating developments to identify themes and collected supporting documentation to enhance the validity and credibility of the findings. The purpose of this step was to triangulate information.

#### Step 5: Analyse and interpret

The study team organised the descriptions of developments in a meaningful way to understand if and how the onset of the COVID-19 pandemic, combined with other factors, produced or impeded broader change in policy or practice domains. Additionally, results were examined across all countries to identify broad lessons learned in maternal health efforts during the COVID-19 pandemic that might provide generalisable insights applicable across countries regarding the process of improving maternal health measurement globally.

#### Step 6: Support use of findings

The study team fed back the case study results to the National Dialogue planning committees in the participating countries to provide them with information pertinent to decision-making, ongoing activities, or future recommendations.

### Data management and analysis

We conducted all FGDs and KIIs remotely via Zoom and recorded. We transcribed the recordings and quality checked the transcripts for accuracy. We analysed all data relating to study aims using an inductive content analysis approach, coding data according to meaning and identifying emergent themes [10]. We used the Dedoose software programme for the qualitative analysis [11].

Our research and reporting follow the criteria recommended in the Standards for Reporting Qualitative Research (SRQR) [12].

### Ethical approval

We obtained ethical approval from the Institutional Review Board at the Harvard T. H. Chan School of Public Health. All participants provided verbal informed consent to participate.

## RESULTS

Mirroring the chaos and conflicting demands of global COVID-19 pandemic on health system and the delivery of essential maternal newborn health care, the study participants observed facing both crises and opportunities. The themes which emerged from the data reflected how COVID-19 exercised conflicting influences on the prioritisation of maternal health, occasionally hindering, yet sometimes facilitating or accelerating developments toward achieving the commitments outlined in the National Dialogues. Here, these emerging issues illustrate the tensions and the complex interplay between confronting the COVID-19 crisis and improving maternal health system performance.

### COVID-19 reinforced existing priorities in maternal health

#### Theme 1.1: Need for continued political commitment

Respondents highlighted that the pressures on the health system exerted by the COVID-19 pandemic generally amplified and reinforced all the priorities identified during the National Dialogues; however, participants emphasised the need for sustained political will in the face of mounting pressures and shifting priorities to drive continued commitment and ensure that progress did not stall during the pandemic.

I think it’s even more relevant now…with the effects that we’ve seen from COVID…we do need to have a very high-level political commitment… because if not, I am afraid, any type of effort will stay at this level, where we’re not moving multi-sectorally… the missing ingredient is where this is seen as a political priority at the highest level. *– Key informant, Mexico*

#### Theme 1.2: Emphasising maternal health as a human right

The extreme health risks and demand for care brought on by the COVID-19 pandemic reinforced the priority identified in some National Dialogues of framing maternal health as a human right for effective advocacy both at the political level and within hospitals and communities. At the political level, both government and non-state actors engaged in advocacy to ensure that the focus on human rights was not lost during the pandemic:

The UN human rights organization contacted us saying that they were alarmed by the increase in maternal mortality due to COVID and then they proposed that we put together a series of seminars to put the issue on the table…Safe motherhood is a human right and implies many more rights, the right to health [and] to have the most advanced care available.* – Key informant, Mexico*

At facility level, there was an emphasis on the health system’s responsibility to treat women in need of maternity care, regardless of their COVID-19 status, as a basic human right.

If [a pregnant woman] was positive for COVID, then it was the responsibility of the hospital to take care of that female. So, these were the changes which were brought into place… *– Key informant, Pakistan*

At community level, respondents emphasised the need to provide people with information about basic rights related to maternal health during COVID as a strategy to empower communities.

Our projects…are advocating for these safe routes of care for women free from COVID infection and for giving communities dignified, concrete and easy-to-understand information.* – Key informant, Pakistan*

#### Theme 1.3: COVID-19 exacerbated known fragilities and challenges in the health system

The COVID-19 pandemic exacerbated health system fragilities previously identified as priorities for improving maternal health and survival. Informants in some settings spoke of broad, overall weaknesses in their systems, such as deficiencies in the organisation of services, while others spoke of the ongoing need to address specific fragilities that were made more pressing during COVID, such as emergency care, distribution of health workers, and availability of midwives.

As a result of the COVID emergency…if [the system is] not really strong, other services are neglected, right? In this case, it is the continuum of maternal reproductive sexual health care…I speak of maternal mortality because it is an issue, but that speaks of the weakness of the health systems that we share with the entire region. In the eyes of people who previously did not see any problem with maternal mortality…now [see] the need to strengthen health services. Primary health care has been highlighted on the national agenda and you know, above all, the continuity of services. – *Key informant, Mexico*

We’ve effectively been facing a public system that is in many ways collapsed, a system that pre-pandemic was already facing fragilities and profound inequalities… Huge deficiencies in the primary level of care and also secondary level. Extremely grave deficiencies in terms of quality of care. All of these deficiencies remain. *– Key informant, Mexico*

When the second wave of COVID hit, it was much more dangerous, and the facilities were busy…people have realized that there are these systemic challenges which was one of the points highlighted from the dialogue. As people spoke about the systems, the health system’s readiness and what needs to be done, especially for the emergency obstetric care. *– Key informant, Mexico*

Informants from all countries also described how COVID-19 exacerbated access constraints that were highlighted in the National Dialogues before the pandemic. These constraints amplified fears of poor treatment that were made worse when combined with the COVID-19 crisis.

During [the EPMM] dialogue, we listened to citizens to understand the challenges that they have had accessing health care services, during the pandemic…there was this anxiety, there were fears for citizens going to health facilities because of the initial news about COVID and the trauma that came with it. So that we listen to citizens, we asked them how difficult it is to access services at health centers when they visit…One of the things that came out were that most of the hospitals were charging them, they were spending more, so the out-of-pocket expenditure increased, and there was huge financial burden on citizens who were seeking care during the pandemic. – *Key informant, Mexico*

In such conditions maternal death increases a lot. Reason being that people are afraid to take care. In India, what is the mindset of the people? In India, the mindset of the people is if they are ill, many times they don't want to go to the hospital, because they know from their own neighborhood and from their relatives that they are not being treated well in the hospital. And you know in COVID times how patients are being treated… but even earlier too, before COVID also…people never liked to… In Indian setting, people think that when you go to the hospital…you even might not come back.* – Key informant, India*

COVID-19 exacerbated existing health systems regarding equity as well. Informants emphasised how the pandemic worsened existing health system inequities, outlining them again as a priority in addressing maternal health. In Mexico, geographic and contextual vulnerabilities combined with COVID-19 to isolate certain population of women.

COVID hospitals were converted from many hospitals where women went for delivery care, but safe routes for pregnant women were not built or established and so continuity of service is cut off and this becomes much more pronounced in isolated populations, the poorest populations, indigenous populations. So, the inequalities…are much more pronounced.* – Key informant, Mexico*

Measurement systems and data quality were often highlighted as critical needs during the National Dialogues, and some informants highlighted how the chaos and pressure under COVID reinforced the need for better data and measurement.

The other thing I see is missing…is bringing more visible data in terms of what the effects of COVID have been on maternal health and maternal mortality. We are in a way working towards that, but we move so slowly that I think making this data available more readily and in a more timely fashion would also help move the agenda to a higher level, politically speaking, and we spoke about this when we met.* – Key informant, Mexico*

We don't yet have the real statistics on how many maternal deaths that [have occurred]. And only when such data is available, we, even the policy sector, will understand the steps that needs to be taken… Some of the indicators and issues that that were discussed in the dialogue also hold true from that context.* – Key informant, India*

### COVID-19 superseded established priorities

#### Theme 2.1: COVID-19 diverted attention from maternal health

Even while amplifying the needs in areas identified as priorities during the National Dialogues, the acute demands of the COVID-19 crisis made governments re-focus their priorities, leading to halts in programmes and advocacy efforts due to limited human resources, funding, and system capacity.

We worked, let's say, with the state health services, and we worked and made strategies and all that, and then when the health emergency came, the States told us to wait, that is, almost, right now, we don’t even now talk because we have to deal with COVID. – Key Informant, Mexico

When COVID came…obviously the priority became COVID. Right? Whatever resources government has had, they were basically being diverted towards COVID.* – Key informant, Pakistan*

Number one priority is COVID-19 and its economic consequences. So, you may see that priority of government changed entirely. It has been very difficult for us to push government to some levels.* – Key informant, Nigeria*

Surveillance tracking actually diverted the dedicated attention that was supposed to given to [maternal health] services.* – Key informant, India*

### COVID-19 facilitated progress towards achieving commitments

#### Theme 3.1: Synergies between COVID-19 and urgent maternal health issues

Despite the challenges brought by the COVID-19 pandemic, respondents described some synergies that emerged between the urgency of the COVID-19 crisis and the ability to bring attention to urgent needs in the realm of maternal health care that were identified at the National Dialogues. Thus, COVID-19 created a platform that activists could use to reinvigorate advocacy for continued investment in maternal health.

One of the meetings that happened about two months ago was [to] resuscitate the basic health care provision fund, because…during the advent of COVID-19, a lot of things went down. Important things were not talked about like about how…the funding [was] supposed to be [dedicated] to respond to key maternal, newborn, and child health [priorities]… So [several advocates] made a video calling on the Nigerian government to pay attention to important things, important things that are ensuring that women and children have access to health and the quality services that they deserve. So, they emphasize the importance that, the fact that access to quality health is the right of the Nigerian citizens, especially mothers and children. And the government should not overlook that because they are responding to a pandemic. So that message was broadcasted on televisions, radio station, and it was huge. it resonated with most advocates in Nigeria, I think that was really a plus for us.* – Key informant, Nigeria*

[The pandemic] put the social determinant in in ways in which people can actually express them. The one that I have actually been quite happy to see…is the fact that health was not something that was considered a governance issue so much, but COVID has led to that and I think civil society organizations have been using that approach to try and, you know, help push government for greater accountability and greater investment into health services.* – Key informant, India*

#### Theme 3.2: COVID-19 response created new programmatic opportunities

Given the impact the pandemic has had on maintaining essential routine care and health services, informants commented that COVID-19 had in some cases created programmatic opportunities to sustain and improve aspects of MCH service delivery and integrate it with the COVID-19 response.

We basically developed what we call a national strategic framework for sustaining Maternal Newborn Child Health and family planning services during the COVID response. So, we kind of developed a stream [of] work in consultation with all the provinces’ key stakeholders through in-depth interviews, focus group discussions, and then having virtual consultations on that framework. And then we were all we were able to kind of integrate what we call the respectful maternity care into the minimum service standards… – Key informant, Pakistan

I think there have been attempts…to establish measures and normative frameworks to operate during the pandemic and without diminishing sexual and reproductive rights of women in the field of maternal health.* – Key informant, Mexico*

The need to understand the pandemic’s rapidly changing epidemiology of and measure its effects and evolution in real time also created a platform for improved data collection for maternal health.

COVID is a really a very challenging situation for Bangladesh, but it is also true that our history says that when our country has faced some challenge, we actually stood up boldly, so COVID is actually giving that opportunity to strengthen our digital system, and you can connect that with the MIS ([health] management information system).* – Key informant, Bangladesh*

### COVID has hindered progress towards commitments

#### Theme 4.1: Pressure of responding to COVID-19 jeopardised quality of care

From a quality-of-care standpoint, COVID-19 exacerbated existing health workforce challenges and brought health workers to the breaking point, leading to lack of attention to commitments related to improving quality of care. Participants were concerned about a lack of both training and supervision because of the demands brought on by the pandemic.

We engaged health workers themselves. And most of them said the challenge was that they were not trained, not given training on…precautionary measures…for tending to patients. And that was a major reason why they were not actually going out to attend to patients.* – Key informant, Nigeria*

The big issue is the lack of supervision, so they are [establishing] norms, but these norms are not applied, are not supervised. There isn’t a system that can guarantee accountability, there is no monitoring and that remains I think it’s worsened during the pandemic.* – Key informant, Mexico*

Despite observations that COVID-19 amplified the need to frame maternal health as a human rights issue, informants detailed how it also created tension between best models of care and upholding women’s rights.

I think that it has also increased obstetric violence, and that needs to be mentioned because, for example, cesarean rates were already incredibly high in the country, which can also be interpreted as a violation of rights of women with COVID. Many hospitals, on their own…and even going against standards that came from the Secretary of Health, began implementing cesarean deliveries as a preventive measure due to the pandemic.* – Key informant, Mexico*

In the case of Oaxaca and Guerrero, that indigenous midwives have been attending many more births than before, and there is no recognition of their work…there are a lot of discriminatory attitudes towards [the] work they do, without considering that it’s the best alternative considering the circumstances. I think that violates human rights. It violates the rights of women to access the services they want and need, and I think that the absence of a good relationship with the health system goes against quality care towards these women in these circumstances because in case of complications, it makes things much harder.* – Key informant, Mexico*

#### Theme 4.2: COVID-19 drained financial resources from maternal health

One way that COVID-19 markedly hindered advancement toward the maternal health commitments across settings was that it drained significant financial resources away from efforts in maternal health.

Last year, 2020, most of the funds have been drawn away, or the commitments were not fulfilled, because of the lack of funds, because they were withdrawn, and they were drawn away towards COVID.* – Key informant, Pakistan*

But from the family planning perspective, all I can tell you is that Pakistan has really run dry on contraceptives in the last one year. Hardly any province… or the central warehouse…procured or was able to procure contraceptives… because of the financial issues, and also because of the some of the regulatory issues.* – Key informant, Pakistan*

The donor landscape has also increased or changed, and so funding priorities and the fiscal landscape is constricting…seriously constricting.* – Key informant, Nigeria*

### What was planned was accomplished

#### Theme 5.1: Government commitment was critical

In some cases, informants described successful achievement of objectives that were set, highlighted, or reinforced during the National Dialogues. Respondents reported notable progress in some areas, even more remarkable given the COVID-19 context. In those cases, a theme that emerged was that continued government commitment was critical to achieving planned activities during the pandemic.

Government is also beginning to make meaningful and consistent commitment and investment in the area of sexual and reproductive health. It is good to tell you that the Federal Government through the Federal Ministry of Health, has just paid their own 2021 counterpart funding in the central basket funding for family planning. If they can at this level, that is [with the] financial constraints and COVID and all that, still bring out money to pay…* – Key informant, Nigeria*

#### Theme 5.2: Progress was slow, but continued

Some respondents emphasised that, despite the pandemic delaying the realisation of the objectives and commitments of the National Dialogues, they managed to raise awareness through advocacy efforts and achieve activities planned during the Dialogues.

The basic health care provision fund is a funding mechanism that Nigeria hopes to use [to] achieve universal health coverage. So that covers…basically maternal, newborn and child health services. So, we have the document drafted…And that document has been validated by the states. And that is, that is one key success for us, because Niger state is like piloting that accountability framework. So, it's, it's a key success for us. I will just conclude to say that the EPMM just was like an eye opener to us. It helped, it helps…identify the important areas for intervention. And that has been the things that we've done, do we have, we have not achieved maximum result? Of course, that's because of the COVID pandemic, the restrictions and all of that, but just set the path for us to success*. – Key informant, Nigeria*

So I think that is I would say the main kind of thing which came out of that dialogue and which was I think, for the first time in Pakistan…people started talking about it and thinking about it…the way it needs to be taken forward…we have been limited in that because of the obvious challenges, which we had related to COVID and also programmatically, but I think these things which I mentioned to you…these are happening in a in a piecemeal manner.* – Key informant, Pakistan*

#### Theme 4.3: Transferring locus of control to local level

In the face of widescale challenges national governments faced due to the pandemic, a theme emerged describing a transfer of the locus of activities to local levels. Participants described being able to make progress at the local level on commitments, even when progress at the national level was stalled.

So that was the time where we basically organized this group…where we came up with provincial discussions and consultations to make sure that the resources with regard to Maternal, Newborn and Child health [were] not diverted towards COVID. And those resources… they stay where they are [allocated].* – Key informant, Pakistan*

Participants also discussed forging deeper connections with communities to fill coverage gaps, while strengthening the connection between communities and the health system.

Working with traditional midwives, and thus being able to make that click, so to speak, between the community, the traditional midwives, and the health services.* – Key informant, Mexico*

### Different activities to address maternal health were prioritised due to COVID-19

#### Theme 6.1: New mechanisms were created to continue efforts

Informants described how they had to adapt to overcome the challenges introduced by COVID-19. These adaptations took several different forms. In some cases, stakeholders described how COVID-19 forced them to pursue new mechanisms for continued coordination. Several comments focused on the ways that COVID had catalysed the use of virtual communication platforms and digital data systems, which in turn created opportunities for increased stakeholder involvement and local participation:

We did a virtual course on dignified treatment and appropriate intercultural services and service providers are taking it.* – Key Informant, Mexico*

I mean, we did at the end of that, that project we did the final dissemination also in Islamabad, where we invited all other provinces and we made the city Government as the host of that event, and it was, again, a hybrid meeting. So, there were people sitting in that Hall and there were people online as well. And the Sindh government basically shared their experiences with other provinces that how they did, how they how what was their experience with regard to this woman involvement and engagement.* – Key Informant, Pakistan*

#### Theme 6.2: Different targets for advocacy emerged

Another common theme highlighted different advocacy targets, strategies, and directions for advocacy emerged as important in the new COVID-19 context.

[We] very strongly advocated [with] the entire group, I mean, the entire group from all the provinces through a very comprehensive consultative process…and kind of reminded the government along with some media coverage…that the resources for Maternal, Newborn, and Child care need to be sustained and increased and not diverted, because of the COVID.* – Key informant, Pakistan*

Today we have an increase in maternal deaths. We’ll see important increases, because there was a loss in the contraceptive prevalence rate for women’s fertility regulation, because they stopped carrying out family planning programs….We already had an important increase in indirect maternal mortality in our state, so these groups of the population who were already seen to be having problems with non-communicable diseases as part of the global burden of disease, and they are continuing with their pregnancy….I think now is a key moment and an important moment to accelerate this momentum that we already had. That’s why I see COVID as a catalyzer, so now perhaps the discourse could include “if we had done this, that wouldn’t have happened,” or “if we’d done that, this wouldn’t have been like that,” “if we’d prepared this way, we could’ve faced those situations. *– Key informant, Mexico*

## DISCUSSION

The onset of the COVID-19 pandemic overturned plans and upended health care systems worldwide, as communities, facilities, health systems, and governments focused on survival. Two to four years after multi-stakeholder National Dialogues were carried out in partnership with government ministries and development partners in seven low- and middle-income countries, the produced plans and intended outcomes were heavily affected. In some cases, new opportunities were presented, while elsewhere, progress towards the agenda outlined in the National Dialogue was halted.

A pattern of paired dualities emerged. For example, for already fragile systems across low- and middle-income countries, the pandemic amplified and worsened health system deficiencies. While this highlighted and brought increased urgency to the maternal health advocacy priorities outlined in the National Dialogues, it also meant those priorities were often superseded by the emergency response to the COVID-19 crisis by health systems staggering under its burden, on top of routine care needs. Similarly, while COVID-19 was seen (in certain ways and in certain contexts) as a catalyser that cleared the path for broad health system improvements upon which maternal health priorities could ride, respondents more often reported that the maternal health agenda items prioritised during dialogues before the pandemic had been greatly delayed or lost in the rush to respond to COVID-related health system emergencies. It has been widely recognised that COVID-19 exposed and exacerbated health disparities globally, while markedly jeopardising maternal health among the most marginalised and disadvantaged populations [13-15]. Our findings underscore the critical risks associated with health inequities and health system deficiencies and the need to address them in order to make maternal health care service delivery more robust to external pressures, such as global pandemics.

Finally, respondents across the countries recounted health system setbacks and associated health-related tragedies – increased maternal mortality, increased mistreatment of women seeking care, widening health inequities, worsening quality of care, and degradation of community trust in health systems. However, they also noted adaptations such as shifting the locus of advocacy and activity from national to sub-national focal areas, catalytic changes in response to the crisis (including the development and improvement of digital communication and data technology), and increased awareness of the importance of identified priorities (including a human rights approach to maternal health). Other studies have noted similar important context-specific adaptations that resulted from COVID-19-related health systems challenges to ensure the continuity of maternal health service delivery, for example, related to supporting community-based efforts and front-line health workers [16] or strategically increasing home-based care (or out of hospital care) delivery led by midwives on home-based care provided by midwives [17]. Calls to ensure that maternal health services uphold women’s and children’s rights despite the urgent pandemic response reverberated globally [18]. As the pandemic begins to subside, country programmes may consider whether and how to institutionalise and optimise the adaptations made during the pandemic to better meet maternal health needs.

Our study has several strengths and limitations. It included a broad range of perspectives from a diverse group of participants in five low- and middle-income countries. Despite this diversity, we were unable to achieve even participation across all countries due to the ongoing COVID-19 pandemic at the time of data collection. Further, our cross-comparative perspective means that we cannot also provide a deep analysis of local context. Many participants in the National Dialogues were at the forefront of COVID-19 response efforts in their countries and were unable to participate in our data collection activities. We believe that engaging a facilitator who was involved in the National Dialogues to conduct the FGDs and KIIs was largely a strength of our study, in that there was already a strong rapport and shared experience built between the participants and the facilitator. In fact, we believe that it may have reduced recall bias, as the facilitator was able to draw on both personal and publicly available documentary artifacts to revisit the proceedings of the National Dialogues during the FGDs and KIIs. Given that the topic of discussion was not particularly sensitive, nor were the participants beholden in any way to the facilitator, we do not believe that this arrangement resulted in any social desirability bias that may have impacted our findings. In particular, the discussion centred largely on contextual observations, rather than personal actions, thus we do not believe that participants would have felt any pressure to respond a particular way when reflecting on progress made or challenges faced. Further, we adapted the outcome harvesting methodology for our study and did not implement it in its complete form. For example, we were not able to map developments against the actors responsible for incremental change or include external substantiators. While these adaptations represent limitations in terms of fidelity to the model, we also believe that they represent a more practical and feasible approach that may be useful for future evaluations to draw on this methodology, given the intensity of inputs required to carry out the methodology in its full form.

## CONCLUSIONS

Our data suggest that the priorities for maternal health system performance improvement to drive toward ending preventable maternal deaths, and the advocacy commitments designed to increase the salience of upstream policy and health system-level determinants of maternal health and survival that were outlined by multiple stakeholders in LMIC prior to the global COVID-19 pandemic largely remain true. If anything, they have been amplified and reinforced by the unprecedented pressure that health systems in these contexts have confronted.
